# Acute Catatonia in Autism Spectrum Disorder: Beyond Pharmacological Treatment

**DOI:** 10.1192/bjo.2026.11790

**Published:** 2026-06-30

**Authors:** Harshini Prasanna Kumar, Mohammed Khan, Ezgi Deniz Yazici, Hafijur Rahman, Yusuf Abdulgafar

**Affiliations:** Birmingham women’s and children’s NHS Trust, Birmingham, United Kingdom

## Abstract

**Aims:**

Catatonia is a neuropsychiatric condition that is frequently under-recognised in children and adolescents and may co-occur with a range of psychiatric and neurodevelopmental disorders, including autism spectrum disorder (ASD). Diagnosis is often challenging due to symptom overlap with neurological and psychotic conditions. In individuals with ASD, catatonia may be precipitated or exacerbated by environmental stressors and disruption to established routines. This case report aims to describe the presentation, diagnostic process, treatment, and longer-term management of catatonia in an adolescent with ASD, with particular emphasis on pharmacological treatment, electroconvulsive therapy (ECT), and psycho-ecological interventions.

**Methods:**

We report the case of a 16-year-old female who presented with a five-month history of mutism, immobility, unsteady gait, and markedly reduced oral intake, resulting in significant weight loss, nasogastric feeding, and hospital admission. Extensive investigations, including CT and MRI brain imaging, EEG, autoimmune encephalitis screening, metabolic testing, chromosomal microarray, and whole genome sequencing, were unremarkable. A lorazepam challenge produced a marginally positive reaction, thus supporting the catatonia diagnosis. The lorazepam dosage was escalated to 12 mg per day, which yielded partial improvement, though this was limited by sedation. Due to the inadequate response, electroconvulsive therapy (ECT) was commenced.

**Results:**

The first six ECT sessions did not produce any improvement; however, from the seventh session onwards, a substantial and sustained recovery was noted, marked by the resumption of speech, mobility, and functional autonomy. Rehabilitation strategies incorporated structured physical activity and social engagement. Despite the initial progress, symptoms later recurred, including psychomotor retardation and confusion. Fluoxetine and quetiapine were subsequently introduced based on collateral history, resulting in partial improvement. A subsequent neurodevelopmental assessment confirmed a diagnosis of ASD, along with learning difficulties.

Following discharge, a relapse occurred several months later, which was linked to stopping lorazepam and less social interaction. When the patient was readmitted, a psycho-ecological approach was emphasised. This approach focused on organising the environment, managing sensory input, establishing predictable schedules, and helping the patient reintegrate socially. All psychotropic medications were stopped due to low efficacy, except lorazepam. Gradual improvement was seen, along with increased involvement in psychosocial activities.

**Conclusion:**

This case highlights the importance of catatonia as a significant but treatable condition in adolescents with ASD. Although lorazepam and ECT were effective for quickly controlling symptoms, lasting recovery depended on psycho-ecological approach with consistent environment, structured routines, family involvement, and ongoing social interaction.

